# Relationship of insulin resistance estimated by triglyceride glucose index to arterial stiffness

**DOI:** 10.1186/s12944-018-0914-2

**Published:** 2018-11-24

**Authors:** Ki-Bum Won, Gyung-Min Park, Sang-Eun Lee, In-Jeong Cho, Hyeon Chang Kim, Byoung Kwon Lee, Hyuk-Jae Chang

**Affiliations:** 1 Division of Cardiology, Ulsan University Hospital, University of Ulsan College of Medicine, 877 Bangeojinsunhwando-ro, Dong-gu, Ulsan, 44033 Republic of Korea; 2 Yonsei Cardiovascular Center, Yonsei University College of Medicine, 50 Yonsei-ro, Seodaemun-gu, Seoul, 120-752 Republic of Korea; 3 Division of Preventive Medicine, Yonsei University College of Medicine, Seoul, South Korea; 4 Division of Cardiology, Gangnam Severance Hospital, Yonsei University College of Medicine, Seoul, South Korea

**Keywords:** Insulin resistance, Arterial stiffness

## Abstract

**Background:**

Insulin resistance (IR) is an important risk factor for subclinical atherosclerosis. This study evaluated the relationship between the triglyceride glucose (TyG) index, which is a simple and reliable surrogate marker for IR, and arterial stiffness.

**Methods:**

This study included 2560 Korean subjects without a previous history of coronary artery disease, stroke, and malignancies who participated in a community-based cohort study. Arterial stiffness was measured using the brachial-ankle pulse wave velocity (baPWV).

**Results:**

All participants were stratified into four groups based on the quartile of the TyG index. The prevalence of metabolic syndrome and diabetes significantly increased with increasing TyG index quartile. The mean baPWV was significantly different among all groups (group I [lowest]: 1421 ± 242 vs. group II: 1480 ± 244 vs. group III: 1534 ± 260 vs. group IV [highest]: 1575 ± 279 cm/s; *p* < 0.001). The TyG index values were correlated with baPWV (*r* = 0.224, *p* < 0.001). Multiple regression analysis showed that age (β = 0.410), male gender (β = 0.051), increased blood pressure (β = 0.266), and TyG index (β = 0.158) were associated with baPWV (*p* < 0.05, respectively). TyG index was independently related to baPWV in both non-diabetics and diabetics.

**Conclusions:**

The TyG index is independently associated with arterial stiffness in a relatively healthy Korean population.

## Introduction

Cardiovascular (CV) disease is a major cause of morbidity and mortality. It is well-established that insulin resistance (IR) is associated with an increased risk of metabolic abnormalities, including hyperglycemia, dyslipidemia, and hypertension [1, 2]. In addition, a number of studies have revealed that IR is one of the most important contributing factors to CV disease [3, 4].

Recently, the triglyceride glucose (TyG) index was used as a simple and reliable surrogate marker of IR. Several studies reported that the TyG index is closely correlated with the homeostatic model assessment of insulin resistance (HOMA-IR) index, which has been traditionally used to measure IR [5–7]. However, there is a paucity of data on the relationship between the TyG index and subclinical atherosclerosis, especially arterial stiffness which has independent prognostic value for the risk of CV events. Moreover, although previous studies suggested the possibility of a somewhat different atherosclerotic change in diabetics compared to non-diabetics, data on the usefulness of TyG index on subclinical atherosclerosis according to diabetic status is currently unavailable. In clinical practice, brachial-ankle pulse wave velocity (baPWV) is used as a simple and reliable tool for the measurement of arterial stiffness because of its high reproducibility. Therefore, the present study evaluated the association between TyG index and arterial stiffness measured using baPWV in a relatively healthy Korean population.

## Methods

### Participants

This is a cross-sectional investigation analyzing baseline data collected for a prospective cohort study. We used the data of 2560 subjects who participated in baseline health examinations for a community-based cohort study in the Seoul area between April 2010 and November 2012. Subjects with a clinical history of cerebrovascular hemorrhage or infarction, neurological abnormalities, or malignancy were excluded from this study. The study protocol was approved by the local ethics committee of our institution, and informed consent for the procedure was obtained from each individual.

All blood samples were obtained after 8 h of fasting and analyzed for triglycerides, high-density lipoprotein (HDL) cholesterol, low-density lipoprotein (LDL) cholesterol, and glucose. Waist circumference was measured at the midpoint between the lower border of the rib cage and the iliac crest. The TyG index was calculated as ln [fasting triglycerides (mg/dL) × fasting glucose (mg/dL) / 2]. Body mass index (BMI) was calculated as weight (kg) / height (m^2^). Metabolic syndrome was defined as when 3 or more of the following were present: (a) blood pressure ≥ 130 mmHg systolic or ≥ 85 mmHg diastolic or anti-hypertensive treatment; (b) HDL cholesterol < 40 mg/dL in males or < 50 mg/dL in females; (c) fasting triglycerides ≥150 mg/dL; (d) abdominal obesity based on waist circumference ≥ 90 cm in males or ≥ 80 cm in females; and (e) impaired fasting glucose, defined as fasting glucose ≥100 mg/dL or established diabetes based on the American Heart Association/National Heart, Lung, and Blood Institute (AHA/NHLBI) definition [8]. Diabetes was defined as either fasting glucose ≥126 mg/dL, a referral diagnosis of diabetes, or antidiabetic treatment.

### Measurement of baPWV

All subjects abstained from beverages or caffeine-containing food for at least 45 min prior to baPWV measurement. After a subject had been resting in the supine position for at least 5 min in a quiet room, blood pressure and baPWV were measured using an automated waveform analyzer (Colin VP-2000, Colin Medical Instruments Corp., Komaki, Japan). Briefly, baPWV was measured in subjects’ bilateral upper and lower extremities using plethysmographic sensor that simultaneously recorded blood pressure, an electrocardiogram, and heart sounds. baPWV was calculated as the length between arterial sites divided by time interval and was measured in both brachial and posterior tibial arteries. The higher value of baPWV measured on either side of each patient was used for analysis.

### Statistical analysis

Continuous variables are expressed as the mean ± standard deviation. Categorical variables are presented as absolute values and proportions. To compare the characteristics among the TyG index groups, one-way analysis of variance was used for continuous variables, and the χ^2^-test or Fisher’s exact test was used for categorical variables, as appropriate. Correlational analysis between the TyG index and baPWV was performed using Pearson’s correlation test. Univariate and multivariate linear regression analysis was performed to identify the association between independent variables and arterial stiffness. Variables with *p* < 0.05 in the univariate analysis were considered confounding variables and entered into multivariate linear regression analysis. All statistical analyses were performed using the Statistical Package for the Social Sciences version 19 (SPSS, Chicago, Illinois), and a *p*-value of < 0.05 was considered significant for all analyses.

## Results

### Baseline characteristics

Table 1 shows the clinical characteristics of the participants. All participants were stratified into four groups based on their TyG index levels. The mean levels of TyG index were 8.7 ± 0.2, 9.2 ± 0.1, 9.5 ± 0.1, and 10.0 ± 0.3 in groups I (lowest), II, III, and IV (highest), respectively. There were significant differences in anthropometric indices, including BMI, waist circumference, and systolic and diastolic blood pressure. The prevalence of metabolic syndrome was 10.0, 19.1, 40.2, and 79.2% and that of diabetes was 7.2, 9.1, 17.2, and 30.6% in groups I, II, III, and IV, respectively.

**Table 1:** Baseline characteristics

	Quartile of the TyG index				*p*
	I (lowest) (*n* = 622)	II (*n* = 658)	III (*n* = 640)	IV (highest) (n = 640)	
Age, years	59 ± 8	60 ± 8	61 ± 8	60 ± 8	< 0.001
Male, n (%)	147 (23.6)	179 (27.2)	224 (35.0)	292 (45.6)	< 0.001
Systolic blood pressure, mmHg	119 ± 14	122 ± 15	124 ± 15	127 ± 15	< 0.001
Diastolic blood pressure, mmHg	71 ± 10	73 ± 9	75 ± 9	77 ± 10	< 0.001
Heart rate, bpm	65 ± 9	66 ± 8	68 ± 10	69 ± 10	< 0.001
Anti-hypertensive drugs, n (%)	195 (31.4)	278 (42.2)	295 (46.1)	326 (50.9)	< 0.001
Smoking, n (%)	114 (18.3)	134 (20.4)	188 (29.4)	259 (40.5)	< 0.001
BMI, kg/m^2^	23.8 ± 2.9	24.5 ± 2.9	25.3 ± 2.9	25.8 ± 2.9	< 0.001
Waist circumference, cm	80 ± 8	83 ± 8	85 ± 8	87 ± 9	< 0.001
Laboratory					
Total cholesterol, mg/dL	191 ± 33	198 ± 36	203 ± 37	205 ± 37	< 0.001
Triglyceride, mg/dL	66 ± 13	99 ± 13	133 ± 21	217 ± 81	< 0.001
HDL cholesterol, mg/dL	64 ± 15	57 ± 13	51 ± 13	45 ± 11	< 0.001
LDL cholesterol, mg/dL	114 ± 29	122 ± 32	128 ± 34	122 ± 35	< 0.001
Fasting glucose, mg/dL	93 ± 11	96 ± 11	101 ± 15	115 ± 31	< 0.001
Creatinine, mg/dL	0.76 ± 0.19	0.77 ± 0.18	0.79 ± 0.18	0.82 ± 0.20	< 0.001
Metabolic syndrome, n (%)	62 (10.0)	126 (19.1)	257 (40.2)	507 (79.2)	< 0.001
Diabetes mellitus, n (%)	45 (7.2)	60 (9.1)	110 (17.2)	196 (30.6)	< 0.001
Anti-diabetic treatment, n (%)	41 (6.6)	56 (8.5)	96 (15.0)	154 (24.1)	< 0.001
TyG index	8.7 ± 0.2	9.2 ± 0.1	9.5 ± 0.1	10.0 ± 0.3	< 0.001

Values are given as the mean ± standard deviation or number (%)

*BMI* body mass index, *HDL* high-density lipoprotein, *LDL* low-density lipoprotein, *TyG* triglyceride glucose

### Relationship between the TyG index and baPWV

**Fig. 1:**
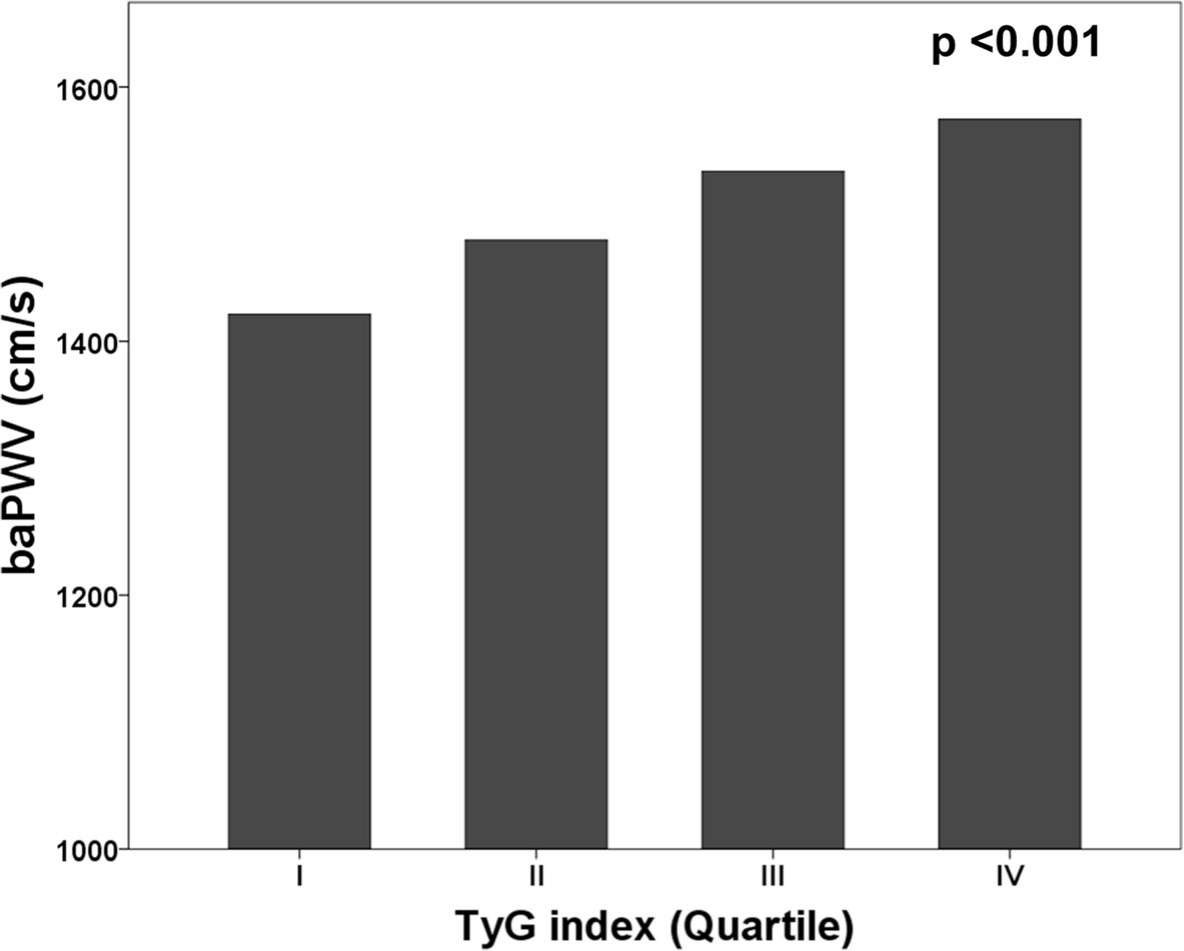
Comparison of baPWV according to TyG index group

**Fig. 2:**
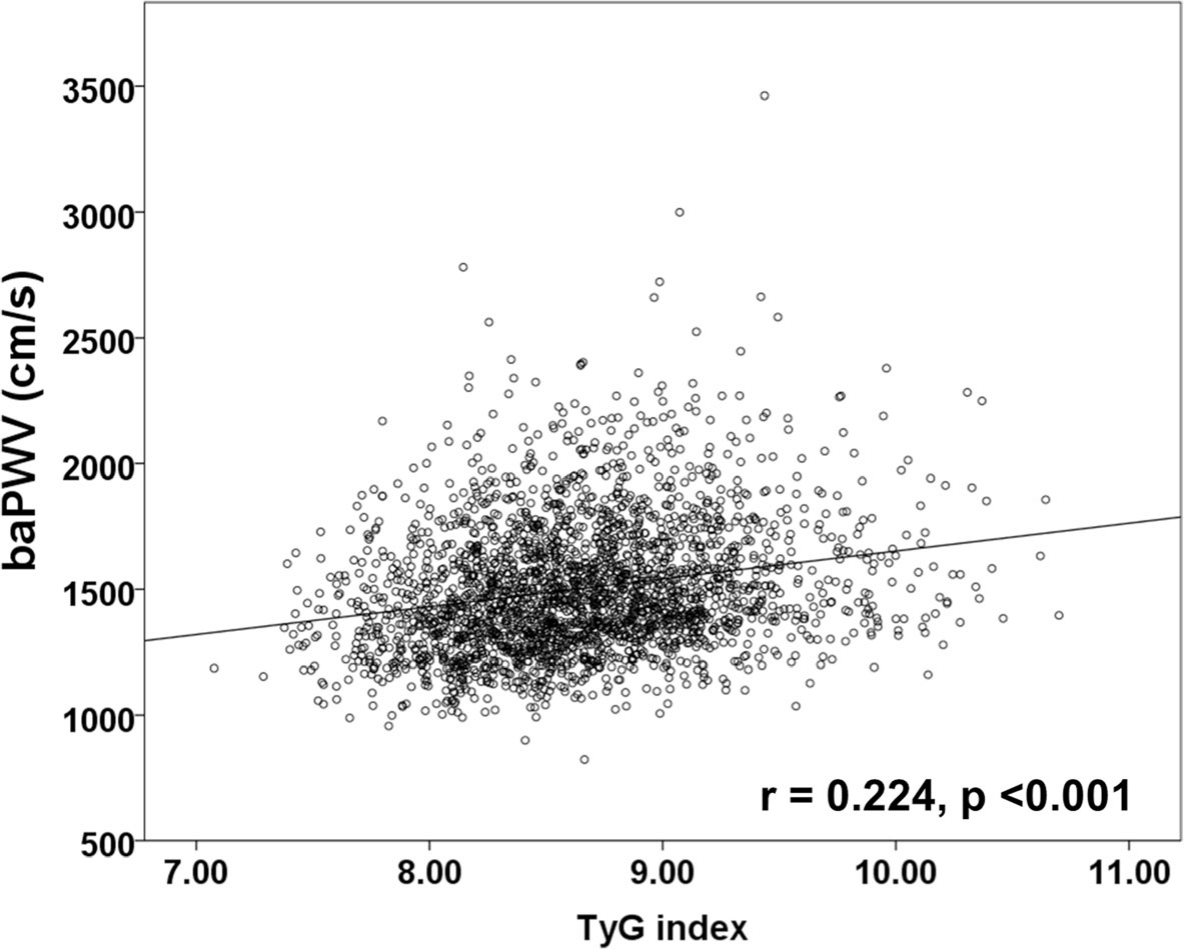
Correlation between TyG index and baPWV

The mean baPWV significantly increased with increasing quartiles of the TyG index (group I [lowest]: 1421 ± 242 vs. group II: 1480 ± 244 vs. group III: 1534 ± 260 vs. group IV [highest]: 1575 ± 279 cm/s; *p* < 0.001) (Fig. 1). The levels of the TyG index were significantly correlated with baPWV (*r* = 0.224, *p* < 0.001) (Fig. 2).

### Association between clinical variables and baPWV

Univariate linear regression analysis showed that age (β = 0.479, *p* < 0.001), male gender (β = 0.137, *p* < 0.001), abdominal obesity (β = 0.083, p < 0.001), increased blood pressure (β = 0.391, *p* < 0.001), decreased HDL (β = 0.057, *p* = 0.004), smoking (β = 0.114, *p* < 0.001), and TyG index (β = 0.224, *p* < 0.001) were significantly associated with baPWV. Multivariate linear regression analysis showed that age (β = 0.410, p < 0.001), male gender (β = 0.051, *p* < 0.043), increased blood pressure (β = 0.266, *p* < 0.001), and TyG index (β = 0.158, *p* < 0.001) were significantly associated with baPWV (Table 2).

**Table 2:** Association between clinical variables and baPWV

	Univariate		Multivariate	
	β	*p*	β	*p*
Age, years	0.479	< 0.001	0.410	< 0.001
Male	0.137	< 0.001	0.051	0.043
Abdominal obesity	0.083	< 0.001	−0.032	0.065
Increased blood pressure	0.391	< 0.001	0.266	< 0.001
Decreased HDL	0.057	0.004	−0.026	0.140
LDL > 130 mg/dL	0.001	0.998		
Smoking	0.114	< 0.001	−0.021	0.396
TyG index	0.224	< 0.001	0.158	< 0.001

*HDL* high-density lipoprotein, *LDL* low-density lipoprotein, *TyG* triglyceride glucose

Increased blood pressure was defined as blood pressure ≥ 130 mmHg systolic or ≥ 85 mmHg diastolic or anti-hypertensive treatment

Decreased HDL was defined as HDL cholesterol < 40 mg/dL in males or < 50 mg/dL in females

### Relationship of TyG index to baPWV according to diabetic status

Multiple linear regression models were analyzed to identify the association between TyG index and baPWV according to the established diabetic status. TyG index was independently related to the baPWV after consecutive adjustment for confounding variables in both non-diabetics and diabetics (Table 3).

**Table 3:** Impact of TyG index on baPWV according to diabetic status

	Non-diabetes		Diabetes	
	β	*p*	β	*p*
Model 1	0.171	< 0.001	0.131	0.004
Model 2	0.161	< 0.001	0.126	0.006
Model 3	0.134	< 0.001	0.125	0.009
Model 4	0.137	< 0.001	0.122	0.011

*HDL* high-density lipoprotein, *TyG* triglyceride glucose

Definitions of increased blood pressure and decreased HDL are present in Table 2

*Model 1* Adjusted for age

*Model 2* Adjusted for age and gender

*Model 3* Adjusted for age, gender, abdominal obesity, increased blood pressure, and decreased HDL

*Model 4* Adjusted for age, gender, abdominal obesity, increased blood pressure, decreased HDL, and smoking

## Discussion

The main finding of present study was that the TyG index was significantly associated with arterial stiffness measured by baPWV after adjusting for other metabolic abnormalities. This result provides evidence that IR has a substantial role in subclinical atherosclerosis in a general population.

The homeostatic model assessment of insulin resistance (HOMA-IR) has been traditionally used to estimate IR [9, 10]. However, insulin levels must be determined to calculate the HOMA-IR index. In South Korea, insulin levels are usually measured for established diabetics. Thus, HOMA-IR is an inconvenient parameter to identify IR in the general population. Recently, several studies reported that the TyG index is closely correlated with HOMA-IR [11, 12]. In addition, some studies suggested that the TyG index had better predictive value for IR than HOMA-IR [6, 13]. Thus, the TyG index is being considered as a simple and useful surrogate marker of IR.

Early detection of atherosclerosis is important for preventing major CV events in the general population. In clinical practice, subclinical atherosclerosis is mostly evaluated at health check-ups with several tools, i.e., coronary artery calcium score (CACS), carotid intima-media thickness, plaque, and pulse wave velocity. Although IR might be a substantial risk factor for the development of CV disease, few studies evaluated the association between IR and subclinical atherosclerosis. In particular, data on the relationship between the TyG index and subclinical atherosclerosis have been limited. Irace et al. reported that the TyG index is strongly associated with carotid atherosclerosis, as assessed by Doppler ultrasonography, after adjusting for traditional CV risk factors [14].^14^ Importantly, they emphasized that the TyG Index is better related to carotid atherosclerosis than HOMA-IR. Kim et al. also reported similar findings that the TyG index is more independently associated with the presence of coronary artery atherosclerosis assessed using CACS than is HOMA-IR in 4319 healthy Korean subjects [15]. In the CRONOS-ADM (Coronary CT angiography evaluation for clinical outcomes in asymptomatic patients with type 2 diabetes mellitus) registry, a higher TyG index is associated with increased risk of coronary artery stenosis in asymptomatic subjects with type 2 diabetes [16]. However, data on the association between the TyG index and arterial stiffness has been limited. The present study investigated the relationship between the TyG index and the arterial stiffness assessed by baPWV in Korean adults without a previous history of major CV events or malignancies. We also found that the TyG index was significantly associated with arterial stiffness after adjusting for confounding factors.

It is well-known that IR is a major characteristic of metabolic syndrome [8, 17]. Additionally, despite the difference in the clinical features of diabetes according to ethnicity [18], IR has a pivotal role in the development of diabetes. Recently, a longitudinal study performed in 2900 non-diabetic adults indicated that the TyG index measured at a single point could be an indicator of the risk for incident diabetes [19]. Participants with TyG index ≥8.8 regardless of obesity had a significantly high risk for diabetes in this study. We confirmed that the prevalence of metabolic syndrome and diabetes significantly increased with increasing quartiles of the TyG index in the present study. Considering that IR has a substantial role in metabolic abnormalities, IR might be an important target to reduce the risk of CV disease. Although previous studies reported the different atherosclerotic change in diabetics compared to non-diabetics, we could identify that TyG index was useful IR parameter for predicting subclinical atherosclerosis in both non-diabetics and diabetics.

This study has some limitations. First, the present study includes only a Korean population. Second, we have not been able to eliminate the possible effects of underlying medications on subclinical atherosclerosis because of the observational design of this study. Third, the impact of IR on arterial stiffness may differ across different age groups. However, it was difficult to perform a sub-analysis of different age groups because none of the cohort study participants were very young. Fourth, we did not measure HOMA-IR because the examination of insulin levels is not usually included in general health check-ups at our institution. However, the close relationship between the TyG index and HOMA-IR was already well-established, as previous described. Fifth, we did not have information on the physical activity of participants. Finally, we did not evaluate the intra- and inter-observer correlation coefficient for the measurement of baPWV. However, it is well-known that the measurement of baPWV is simple and reliable for identifying arterial stiffness because of its high reproducibility [20, 21]. Despite these limitations, we could identify the independent impact of the IR estimated by the TyG index on arterial stiffness, which is an important marker of subclinical atherosclerosis.

In conclusion, the TyG index was independently associated with arterial stiffness measured by baPWV in a relatively healthy Korean population. This result suggested that that IR has a substantial role in subclinical atherosclerosis and might be an important target to prevent major CV disease.

## Abbreviations

baPWV: Brachial-ankle pulse wave velocity
BMI: Body mass index
CVD: Cardiovascular disease
HDL: High-density lipoprotein
HOMA-IR: Homeostatic model assessment of insulin resistance
IR: Insulin resistance
LDL: Low-density lipoprotein
TyG: Triglyceride glucose
